# A pre-trained convolutional neural network with optimized capsule networks for chest X-rays COVID-19 diagnosis

**DOI:** 10.1007/s10586-022-03703-2

**Published:** 2022-08-23

**Authors:** Lobna M. AbouEl-Magd, Ashraf Darwish, Vaclav Snasel, Aboul Ella Hassanien

**Affiliations:** 1 Computer Science Department, Misr Higher Institute, Mansoura, Egypt; 2 Faculty of Science, Helwan University, Helwan, Egypt; 3 VSB-Technical University of Ostrava, Ostrava, Czech Republic; 4 Faculty of Computers and AI, Cairo University, Giza, Egypt; 5 Scientific Research Group in Egypt (www.egyptscience.net), Giza, Egypt

**Keywords:** COVID-19, Coronavirus, Convolution neural networks, Capsule Neural Networks, VGG16, Gaussian optimization method

## Abstract

Coronavirus disease (COVID-19) is rapidly spreading worldwide. Recent studies show that radiological images contain accurate data for detecting the coronavirus. This paper proposes a pre-trained convolutional neural network (VGG16) with Capsule Neural Networks (CapsNet) to detect COVID-19 with unbalanced data sets. The CapsNet is proposed due to its ability to define features such as perspective, orientation, and size. Synthetic Minority Over-sampling Technique (SMOTE) was employed to ensure that new samples were generated close to the sample center, avoiding the production of outliers or changes in data distribution. As the results may change by changing capsule network parameters (Capsule dimensionality and routing number), the Gaussian optimization method has been used to optimize these parameters. Four experiments have been done, (1) CapsNet with the unbalanced data sets, (2) CapsNet with balanced data sets based on class weight, (3) CapsNet with balanced data sets based on SMOTE, and (4) CapsNet hyperparameters optimization with balanced data sets based on SMOTE. The performance has improved and achieved an accuracy rate of 96.58% and an F1- score of 97.08%, a competitive optimized model compared to other related models.

## Introduction

Coronavirus (COVID-19), which originated in December 2019 at Wuhan Province of the People's Republic of China, presents a severe and deadly threat to health worldwide. COVID-19 has infected more than 8,963,350 people in 188 countries, and the aggregate of people who died is increasing [1]. There are several medical ways of diagnosing COVID-19. Chest radiography is the preferred imaging method for people infected with COVID-19 because it is easily accessible, cheap, and easy to clean and disinfect [2]. The most common radiographic findings are the airspaces' opacity, described as a fusion, or, less commonly, the Earth's glass's opacity. The distribution of COVID-19 is often binomial, circumferential, and inferior [2].

Convolutional Neural Network (CNN) has important medical image analysis and processing applications. The results of CNN show that it can translate image data to a precise and expected output [3, 4]. Furthermore, Deep learning-based chest X-rays can also diagnose diseases faster than traditional methods. Several authors used CNN for medical applications in their research papers. For instance, authors in [5] and [6] used the CNN-based CheXNet model for chest diseases.

Some researchers use pre-trained CNN. For example, in [7], the authors used pre-trained ResNet-50 architecture named COVID ResNet. The input layer, the group of hidden layers, and the output layer are the three primary layers of a CNN. Convolutional, pooling, fully linked, and normalization layers are also included in the hidden layers. When it comes to image-related operations, CNN excels. They do, however, have some inherent limitations and flaws. CNN, for example, fails to capture relative spatial and orientation relationships and is easily confused by changes in image orientation or pose. The max-pooling layer is critical because it downsamples the data and decreases the spatial information given to the next layer. On the other hand, the max-pooling layer has a disadvantage for CNN because it cannot convey spatial hierarchies across various objects. This flaw causes invariance, and the pose and spatial.

Although CNNs perform well in dealing with images, they still have a set of shortcomings. The aggregation process used in convolutional neural networks suffers from losing valuable information when using aggregation layers. In addition, they require huge amounts of data for learning. Layering in a CNN reduces spatial resolution, and the networks' output never changes even with a small amount of change in the input. It cannot be directly related to the relationship of parts and requires additional components. This is where Capsule Networks comes into play and overcomes all the drawbacks of CNN. Capsule networks (CapsNet) can fetch spatial information to overcome information loss in aggregations [8].

CapsNet is a novel type of neural network presented in [9], which introduced a "capsule" concept. A capsule is a bunch of neurons. Each layer in a capsule network has several capsules. The Capsule's outputs have different properties of the same entity. The traditional CNN is based on the vision system's use of the same knowledge at all locations within an image. This is often accomplished by linking feature detector weights to make features learned in one location available in others. Convolutional capsules extend knowledge sharing across sites to include the part-whole relationships that characterize the familiar form. This is achieved when a layer's position matrix is multiplied by a trainable viewpoint static transformation matrix that can learn to represent part-to-total relationships, capsule votes for the position matrix of several capsules above it in that layer [10].

The Capsule has hyperparameters that affect its algorithm's complexity and accuracy of a given problem [11]. These hyperparameters are the number of routing and capsule dimensionality. Many optimization algorithms can be used to get optimal hyperparameter values.

This paper proposes a new model that uses pre-trained CNN VGG16 with a CapsNet to detect COVID-19. The accuracy of the proposed model is enhanced by using the Gaussian optimization process to tune the hyperparameters of the Capsule neural networks (CapsNet).

The main contribution of this paper is summarized as follows.

- Handling the balancing and the small number of images on the benchmark database as two problems may impact the detection and classification results
- Pre-trained the model is used for deep feature extraction using VGG16 as input for the CapsNet.
- Detection of COVID-19 detection model based on pre- train VGG16 with Capsule Networks
- Applying Gaussian optimization for CapsNet hyperparameter optimization

This paper is organized as follows. Section 2 reviews related works. Section 3 focuses on the preliminaries and basics, whereas Sect. 4 presents the materials and methods. Furthermore, Sect. 5 describes the experimental results and analyzes the results and performance of the proposed model. Finally, Sect. 6 has the conclusion and highlights future work.

## Related works

Artificial intelligence has been used to recognize and classify lung diseases in recent decades. Research has varied between image feature extraction, suitable image recognition, and disease identification classifiers. For example, Patil in [12] classified Lung cancer based on a texture features extraction and the backpropagation neural network. The retrieved features were average grey level, standard deviation, smoothness, the third moment, uniformity, and entropy. Furthermore, an accuracy of 83 percent was obtained. The authors used multilayer, probabilistic, learning vector quantization, and generalized regression neural networks to achieve a comparative chest illness diagnosis [13]. They demonstrated that the probabilistic neural network performed better.

The spread of the coronavirus has been a great danger since its outbreak in Wuhan in December 2019. This virus has a real worldwide disastrous impact, as the number of deaths reached 2,624,677 and confirmed cases 118,268,575 in about 223 countries, according to the reports of the World Health Organization on March 12, 2021 [14]. This motivated many researchers to identify this dreaded disease and limit its spread.

Pereira et al. in [15] created RYDLS-20, a database of CXR images of pneumonia and healthy lungs. They used multiclass and hierarchical classification and resampling algorithms to deal with an unbalanced data set. They compare and use different feature extraction algorithms for extracting features from the image, such as binarized statistical image features (BSIF), local binary patterns (LBP), local directional number pattern (LDN), elongated quinary patterns (EQP), local phase quantity (LPQ), and Basic Oriented Image Features OBIF. The suggested technique yielded an average F1 score of 0.65 using a multiclass approach in the hierarchical classification scenario and an F1 score of 0.89 for COVID-19 identification.

Tej Bahadur Chandra et al. [16] employed a variety of ways to extract features from images and then used binary grey wolf optimization to select the best ones. Their study performs classification in two phases. The first phase distinguishes between normal and abnormal chest images. The second phase (phase II) distinguishes pneumonia and Covid-19 chest images. Additionally, the voting-based classifier ensemble is used for classification. The majority vote-based classifier ensemble in phase (I) gave 98.062% accuracy and 98.55for the F1 score, and phase II gave 91.32% accuracy and 91.73 for the F1 score. Furthermore, the majority vote-based classifier ensemble gave an overall accuracy of 93.41%.

CNN has been used in many types of research for detecting the COVID-19. For example, Asmaa Abbas et al. [17] used a deep CNN called decompose, transfer, and compose to detect COVID-19 X-ray pictures (DeTraC). They demonstrated DeTraC's competence in detecting COVID-19 with a 93.1% accuracy. Any anomalies in the picture dataset are dealt with utilizing a class decomposition approach by DeTraC.

O. M. Elzeki et al. [18] proposed a CXR COVID Network (CXRVN) network. CXRVN is a compact architecture based on a single fully-connected node. The CXRVN uses Mini-batch gradient descent and Adam optimizer. The authors used three datasets to test their model. Dataset-1 comprises two class labels and 50 X-ray images, and the test accuracy was 92.85%. Additionally, Dataset-2 comprises two class labels and 455 X-ray images with 96.70% accuracy. Furthermore, Dataset-3 comprises three class labels and 603 X-ray images, and the accuracy was 91.70%. Moreover, they used generative adversarial networks (GAN) augmentation, giving 96.7% accuracy in Dataset-2 for two classes and 93.07% in Dataset-3 for three classes. The average accuracy reached 94.5%.

M.Nour et al. [19] used CNN for extracting discriminative features and different machine learning approaches for classification. The hyperparameters of their models were optimized using the Bayesian optimization algorithm. The support vector machines classifier ensured the most efficient results with 98.97% accuracy and 96.72% F1-score.

Some researchers used pre-train CNN models and transferred learning for COVID-19 detection to get more accurate results. M. M. Rahaman et al. used deep transfer learning to compare 15 pre-trained CNN models. The VGG19 had an F1 score of 0.90 and an accuracy of 89.3% [20].

Arun Sharma et al. [21] Used transfer learning to build AI-based classification models, CXR pictures depicting the investigated diseases may be accurately classified. They performed 25 different augmentations on the original pictures to increase the dataset. They then used a transfer learning method to train and test their models. After training 286 images in each of the two best models, combining them produced the maximum prediction accuracy for the normal, COVID, non-COVID, and pneumonia/tuberculosis shots.

For binary (COVID vs. No-Findings) and multiclass classification (COVID vs. No-Findings vs. Pneumonia), Tulin Ozturk et al.[22] introduced the DarkNet model. Binary classification accuracy was 98.08%, while multiclass classification accuracy was 87.02% for their model.

In [23], D. Ezzat et al. proposed an optimized hybrid CNN. The used CNN architecture is DenseNet121, and the optimization algorithm is the gravitational search algorithm (GSA). The GSA is used to determine the most appropriate values for the hyperparameters of the DenseNet121 architecture. Their accuracy reached 98.38%

In [24], Hossein Abbasimehr et al. suggested a method to forecast COVID-19 time series using three deep learning techniques: (1) LSTM, (2) gated recurrent units, and (3) CNN. The suggested strategy considerably increases the performance of LSTM and CNNs in terms of symmetric mean absolute percentage error and root mean square error measurements.

Table 1 shows the relevant work on COVID-19 diagnosis from chest X-ray radiographs; we can see from this Table that the performance of the majority of the pieces is poor, especially in multiclass work, except Nour et al. [19], where the f1-score is lower than the accuracy. As a result, we were motivated to increase the accuracy of multiclass classification by employing a capsule network, which is concerned with collecting the pose and spatial interactions between image pixels.

**Table 1:** Summary and analysis of the related works

Paper	Method	Classes	Performance
Rodolf M.Pereira et al. [15]	Use different feature extraction algorithms	Multiclass	The multiclass F1-score was 0.65, and the hierarchical classification F1-score was 0.89
M. M. Rahaman et al. [20]	VGG19	Multiclass	Accuracy was 89.3%, and F1 score was 0.90
Asmaa Abbas et al.[17]	Deep CNN	Binary class	Accuracy was93.1%
Tej Bahadur Chandra et al. [16]	Used different feature extraction techniques and used binary gray wolf optimization for feature selection. Also, used voting-based classifier ensemble is used	Distinguishes between normal and abnormal chest images and distinguishes between pneumonia, and the Covid-19 chest	Phase (I) gave 98.062% accuracy and 98.55 for the F1-score, and phase II gave 91.32% accuracy and 91.73 for F1 score The majority vote-based classifier ensemble gave an overall accuracy of 93.41%
O. M. Elzeki et al. [18]	proposed a network architecture called CXR COVID	Multiclass	- Accuracy was 96.7% - Accuracy was 93.070%
M.Nour et al. [19]	Suggested model based on CNN	Multiclass	Accuracy was 98.97% and an F1-score was 96.72%
Tulin Ozturk et al.[22]	proposed the DarkNet model	binary classification multiclass classification	The binary class accuracy was 98.88%, while the multiclass accuracy was 87.02%
Dalia [23]	DenseNet121	The gravitational search algorithm is used to determine the best values for the hyperparameters of the DenseNet121 architecture	Accuracy 95%

## Preliminaries

### Capsule neural networks

CNN is currently used in many applications and has shown satisfactory results in these applications' classification and prediction processes. However, CNN performs poorly in recognizing position, texture, and distortions of an image or parts of an image. This implies that the CNNs are invariant. The pooling phase in CNN may produce invariance. CNN's are not equivariant and thus lack equivalence. Furthermore, some images' features are lost due to the pooling operation on CNN.

**Fig. 1:**
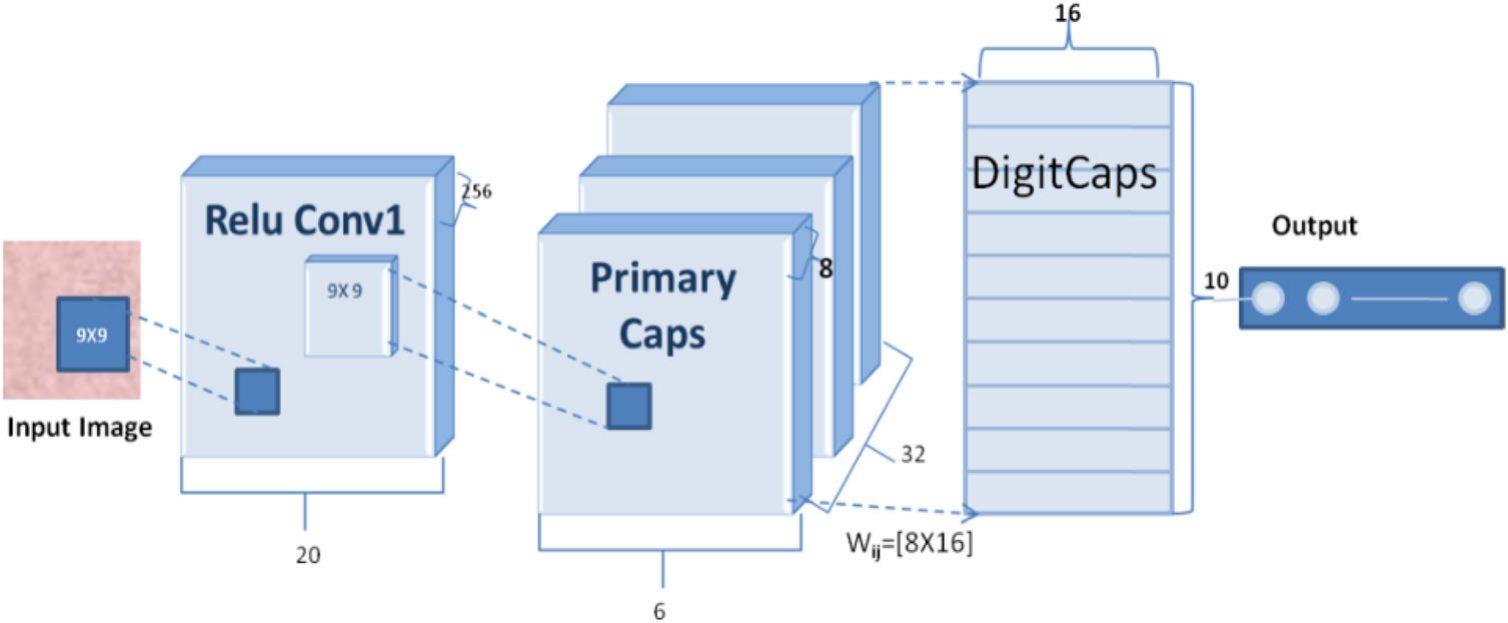
Capsule network architecture

Consequently, CNNs are being supplanted by capsule networks. Unlike CNN, capsules are equivariant and consist of a network of neurons that input and output vectors rather than the scalar values. This capsule property allows it to learn the image's deformations, viewing conditions, and features [25, 26]. Capsule networks are made up of layers of capsules. Each Capsule comprises a collection of neurons whose output represents a distinct aspect of the same feature. CapsNet was introduced in [9], where the Capsule is defined as a set of neurons with activity vectors representing instantiation parameters and the length of the vector, signifying the likelihood of the feature existing. The CapsNet model used in this paper consists of 3 main successive layers, as shown in Fig. 1, including the convolutional layer, primary capsule (PC), and class capsule layers named digit caps layer [9].

The first layer (convolutional layer) is responsible for image feature extraction, where the image's pixel is converted to spatial information. The output of the convolution layer enters the PC layer. The PC layer may act as though the rendering process was reversed. An image's information can be divided into numerous units under several channels to generate a vector of reserved spatial data for each unit. It reaches the class capsule's next layer of neurons. This innovative network structure replaces the pooling layer in a normal convolutional network, reducing information loss significantly [9, 27, 28].

An overview of how the capsule network works is as follows. Layer l, each Capsule *i* has an activity vector o_i_ that encodes spatial information in instantiation parameters. The ith lower-level capsule's output vector o_i_ is supplied to all capsules in the next layer l + 1. At layer l + 1, the jth Capsule will receive o_i_ and locate its product using the weight matrix W_ij_. The $\hat{o}$_j|i_ vector transforms Capsule j at level l + 1 by Capsule *i* at level l. $\hat{o}$_j|i_ is a PC's prediction vector showing how much the primary Capsule *i* contributes to class j.

1 $$\hat{o}_{j\left| i \right.} = W_{ij} o_{i}$$

One main capsule i's prediction for class capsule j is made by multiplying its prediction vector by its coupling coefficient, which measures the degree of agreement between the two caps. They are linked as long as there is a significant degree of agreement between the two capsules. As a result, the coupling coefficient will increase rather than decrease as would occur if the opposite occurred.

To find the squashing function candidates, a weighted sum (ws_j_) of all these individual PC predictions for the class capsule j is produced (q_j_).

2 $${\text{ws}}_{{\text{j}}} = \sum\nolimits_{i = 1}^{N} {c_{ij} \hat{o}_{j\left| i \right.} }$$

3 $${\text{q}}_{{\text{j}}} = \frac{{\left\| {{\text{ws}}_{{\text{j}}} } \right\|^{2} }}{{1 + \left\| {{\text{ws}}_{{\text{j}}} } \right\|^{2} }}\frac{{{\text{ws}}_{{\text{j}}} }}{{\left\| {{\text{ws}}_{{\text{j}}} } \right\|}}$$

4 $${\text{c}}_{{{\text{ij}}}} = \frac{{\exp ({\text{b}}_{{{\text{ij}}}} )}}{{\sum\nolimits_{{\text{k}}} {\exp ({\text{b}}_{{{\text{ij}}}} )} }}$$

The squashing function, like a likelihood, assures that the length of the output from the Capsule is between 0 and 1. The q_j_ from one capsule layer is passed on to the next capsule layer, where it is treated the same way as before. The c_ij_ coupling coefficient ensures that the level l prediction of I is linked to the layer l + 1 prediction of j. The dot product of ô j|i and qj is obtained during each cycle, and c_ij_ is updated. The vector values of each Capsule may be thought of as a combination of two numbers: a probability indicating the presence of the feature encapsulated by the Capsule and a set of instantiation parameters that can be used to explain layer consistency. The term "relevant path by agreement" comes from the fact that when lower-level capsules agree on a higher-level layer capsule, they "construct a part-whole" relationship demonstrating path relevance. Dynamic routing-by-agreement is the name for this approach [9, 10, 25].

Capsule networks suffer from expensive computational methods, yet numerous routing layers increase training costs and inference time because of the complexity of the network [29]. Researchers in [24] have, on the other hand, demonstrated that the prediction time is significantly shorter than that of other deep learning techniques. In this research, the optimization strategy focuses on the number of routing to get high performance with minimal complexity.

### VGG16 architecture

**Fig. 2:**
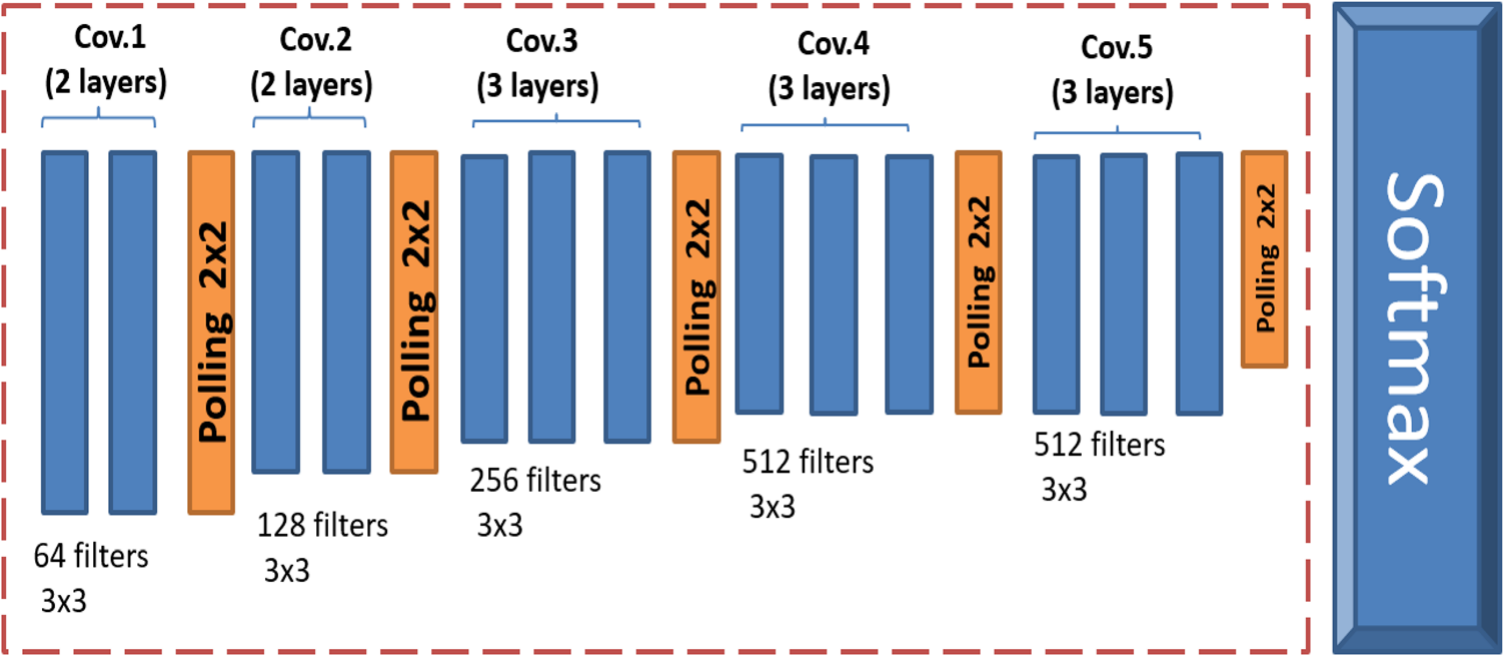
The architecture of the pre-trained VGG16

VGG16 is a CNN model introduced in [30]. VGG-16 includes 16 layers, 13 of which are convolutional and three of which are fully linked. Five blocks comprise the convolutional layers. The model uses only filters of size 3 × 3 with one stride for convolutions and 2 × 2 pooling with two strides in all layers, resulting in a homogenous and smooth architecture. The pre-trained VGG16 model can classify images into 1000 object categories with million images. It got an accuracy of about 92.7% in the accuracy test for the ImageNet dataset—VGG16 improves other CNN models like AlexNet. The NVIDIA Titan Black GPU has been used for training the VGG16 [30]. Figure 2 shows the VGG16 architecture.

### Gaussian optimization algorithm

The Gaussian process (GP) is specified by its mean and covariance functions. It can be written as:

5 $$F(x) \sim g \cdot p(m(\lambda ),{\text{ K}}(\lambda , \, \lambda^{ \sim } ))$$

where m(λ) denotes mean, and K denotes covariance [31].

The Bayesian model is a simple example of a GP, and the Bayesian optimization method is one of the general optimization methods; the optimization method is an iterative algorithm. A probabilistic surrogate model element of the Gaussian optimization approach and an acquisition function determining the next point to be assessed are included. The surrogate model fits all target function observations thus far in each cycle. The assemblage function estimates the utility of candidate nodes using the probabilistic model's prediction distribution. Rather than assessing costly efforts, the importance of acquisitions is calculating and optimizing them [26, 31]. The expected improvement (EI) is based on Eq. (6) and is the acquisition function.

6 $${\text{E[I(}}\lambda {\text{)] = E[max(f}}_{{{\text{min}}}} - {\text{Y, 0)]}}$$

where (EI) is computed in the closed-form if the prediction of the Model Y at configuration λ according to a normal distribution is defined using Eq. (7).

7 $$E[I(\lambda )] = ({\text{f}}_{\min } - \mu (\lambda ))\Phi \left( {\frac{{{\text{f}}_{\min } - \mu (\lambda )}}{\sigma }} \right) + \sigma \phi \left( {\frac{{{\text{f}}_{\min } - \mu (\lambda )}}{\sigma }} \right)$$

where φ(·) is the standard normal density, Φ(·)s the standard normal distribution function, and *f*_*min*_ is the best-observed value.

### Synthetic minority over-sampling technique (SMOTE)

Machine learning algorithms are usually evaluated based on their predictive accuracy. The dataset is imbalanced if the classes are not roughly equally represented. When the data is uneven, this is ineffective. The data-level technique aims to rebalance the minority and majority classes by modifying the data. This can be done by either removing some examples from the majority class (under-sampling) or increasing the number of cases from the minority class (over-sampling) (over-sampling). SMOTE is an over-sampling method that uses "synthetic" instances rather than replacement over-sampling to over-sample the minority class [32].

## The proposed pre-trained CNN with optimized CapsNet for chest X-Ray COVID-19 diagnoses

**Fig. 3:**
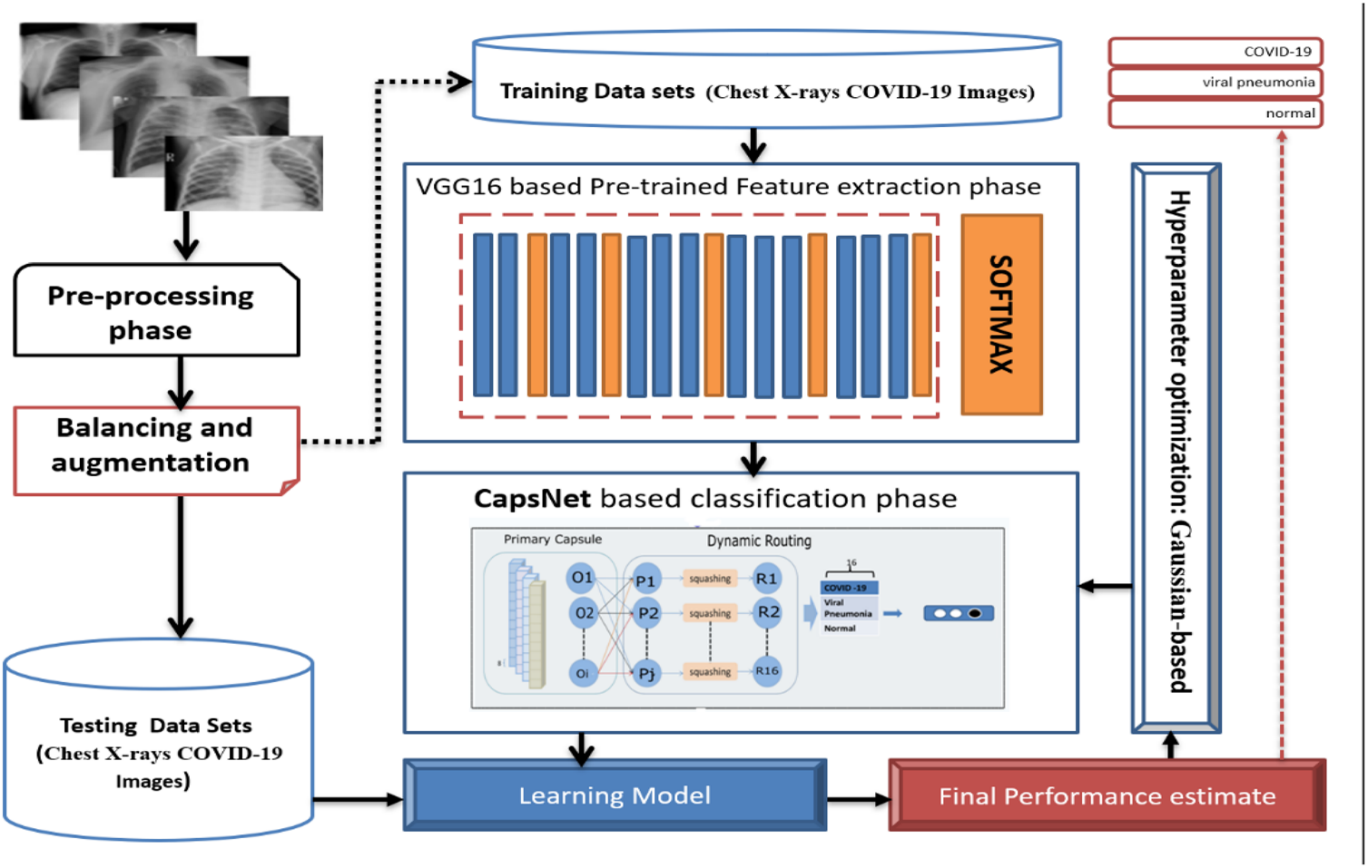
The proposed COVID-19 prediction model using Optimized CapsNet

The proposed COVID-19 detection model mainly includes four phases, as shown in Fig. 3: data preprocessing, pre-trained, classification and optimization, and evaluation phases. Each of these phases is explained in the following subsections.

### Data preparation phase

Overfitting occurs when a network learns a function with a high variance to model the training data successfully. This phase implemented three key steps: splitting data into training and testing sets, data augmentations, and balancing the dataset. Due to its novelty, we discovered two issues with the entire dataset. The first problem is that there are few data points, and the second is that the data is unbalanced. The medical dataset only contains a few photos, whereas deep learning algorithms require a large amount of data to avoid overfitting.

There are many ways and techniques to avoid overfitting (raised from the small dataset); one of them and the most used is data augmentation. We used the image data augmentation methods, such as rotating right with 30 degrees, left with 30 and + 90 degrees up and horizontally about Y-axis, and shear in this work.

We employ two approaches to deal with the unbalanced dataset. The first one is based on the class weight based on costly errors. The cost error value will be included in the probability for each class. The second approach is using SMOTE technique based on over-sampling. It involves multiplying select points from the minority class to broaden their origin. Another issue in the preprocessing phase is splitting the dataset into training and testing datasets with 70% and 30% ratios.

### Pre-trained and CapsNet optimization phases

The training process is divided into the following three steps.

**Fig. 4:**
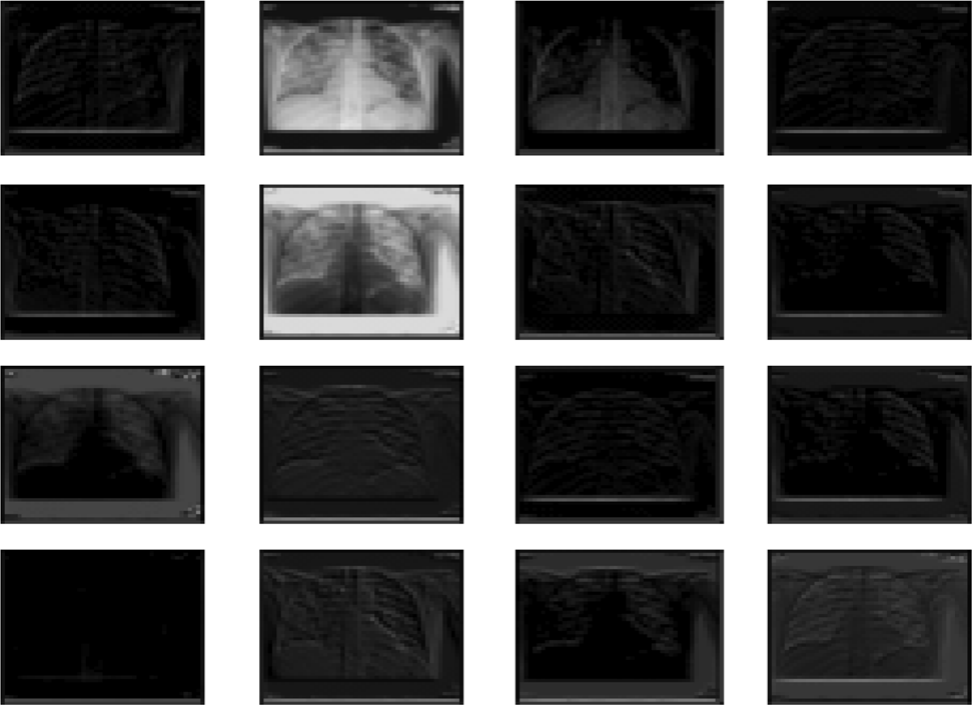
Visualization of VGG16 output

*Step (1)*: Pre-train CNN VGG16 Architecture: generally, the elementary features can be extracted on CNN. The elementary feature may be edges and corners. The extracted features are aggregated in the next layers to detect higher-order features. CNN has another essential property; this property shares weights meaning that similar feature detectors are utilized for the whole object. CNN has many layers known as "sub_sampling" layers. The "sub_sampling" layers depend on the fact that the features' exact location is useful and destructive since this dataset tends to vary for different images or objects [33]. Transfer learning from a pre-trained model like VGG16 extracts the in-depth features. The image is input to the pre-trained network, and the activation values for different layers are stored and utilized as features [34]. The VGG16 consists of five blocks that help get similar features instantly. So, VGG16 will be used on the recent new architecture called CapsNet introduced in [9]. Figure 4 shows the visualization of VGG16 output.

*Step (2)*: Capsule network (COVID-caps): The X-ray image of COID-19 differs from the normal image because it contains white spots on the lung; also, it differs from the viral pneumonia image due to the location, whereas in covid-19, these spots spread at the bottom of the lung more, i.e., the area of the spots contributes to identifying COVID-19. Instead of neurons CapsNet is made up of capsules. As defined in [35], the Capsule can be a collection of neural networks. It could carry out complex internal calculations on their inputs and store the results in a tiny vector. The Capsule records the relative position of the item, and if the object's pose changes, the output vector orientation also changes. CapsNet is made up of several layers. The first layer is called PCs, consisting of individual capsules that each receives a small portion of the receptive field as input and attempt to determine the pose of a specific pattern. The Capsule output is a vector, and a dynamic routing mechanism was employed to ensure that the result was sent to the appropriate parent in the layer, which could be deduced.

The CapsNet architecture consists of two layers.

(i) The PC layer is the first layer of the CapsNet that follows the pre-train model. It is a convolutional capsule layer that contains 32 channels of convolutional 10 D capsules. Each PC has ten convolutional units with a (9 × 9) kernel, and the number of the strides is 2.
(ii) COVID-caps capsule layer contains three capsules, denoted as "COVID-caps," and one candidate of lung disease (COVID-19, viral pneumonia, or normal). The COVID capsule layer consists of 10 capsules, each representing a particular class of the lung disease dataset with three. The initial dimension used for these capsules is 16. Computed COVID-caps capsules' output calculates the predicted output vectors for each primary COVID-caps capsule pair and implements the route by agreement algorithm.

Margin loss for lung diseases is the length of the instantiation output vector representing the probability of the respective Capsule's entity existence. For every disease_kind Capsule Co, the margin loss is separate and is given in Eq. (8). The disease class Co has the most prolonged vector output if the lung disease is present in the input image.

8 $${\text{L}}_{{{\text{Co}}}} = {\text{T}}_{{{\text{Co}}}} \max (0,\,{\text{mr}}^{ + } - \left\| {{\text{V}}_{{{\text{Co}}}} } \right\|)^{2} + \lambda (1 - {\text{T}}_{{{\text{Co}}}} )\max ((0,\left| {\left| {{\text{V}}_{{{\text{Co}}}} } \right|} \right| - {\text{mr}}^{ - } )^{2}$$

The value of T_Co_ is 1 if a lung disease of class Co is present, and in this study mr^+^  = 0.9 and mr^−^ = 0.1. λ is a regularization parameter that stops learning from shrinking all lung disease capsules [10].

**Fig. 5:**
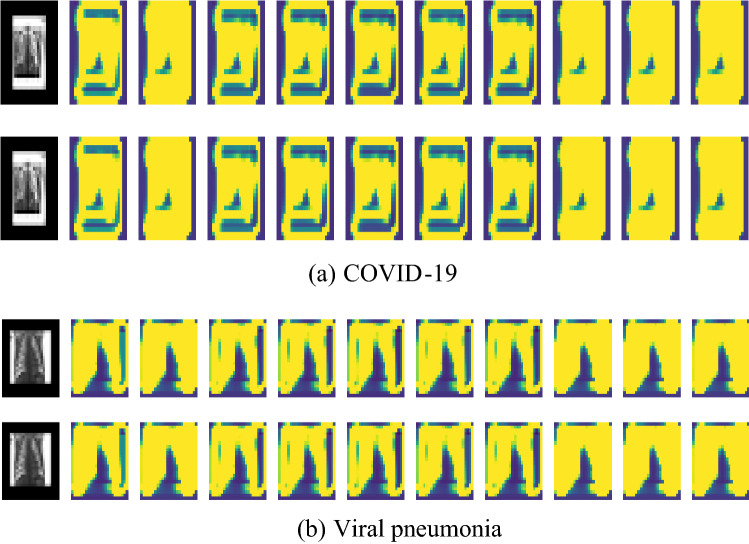
CapsNet visualization. **a** COVID-19. **b** Viral pneumonia

Figure 5 presents the visualization of the CapsNet. The disease spots are visible in Fig. 5a for Coivd-19 disease and Fig. 5 (b) for viral pneumonia.

The accuracy is computed as the correctly identified lung disease ratio by the total number of lung diseases.

9 $${\text{Accuracy = }}\frac{{\Sigma \,{\text{Correct}}\,{\text{identified}}\,{\text{lung}}\,{\text{diseases}}}}{{{\text{Total}}\,{\text{number}}\,{\text{of}}\,{\text{lung}}\,{\text{diseases}}}}$$

*Step (3)*: CapsNet hyperparameter optimization: Hyperparameter optimization is a way to find a D- dimension hyperparameter setting x that minimizes the validation loss/error f of the CapsNet learned with. The function f maps a hyperparameter choice $x$ of *G* configurable hyperparameters to a CapsNet algorithm's validation error with learned parameters [36]. Optimizing f, as shown in Eq. 10, suggests a solution for finding out the optimal hyperparameters automatically:

10 $$\begin{gathered} min_{{x \in R^{G} }} f(x,\theta ;S_{{val}} ) \hfill \\ s.t.\theta = \arg \min _{\theta } f(\theta ;S_{{{\text{train}}}} ) \hfill \\ \end{gathered}$$

Solving the problem in Eq. (10) is quite challenging due to the incredible complexity of the function *f*. Where S_train_ denotes the training dataset, and ${S}_{val}$ represents the validation dataset. The learning process reduces the training loss/error, and the value of *x* is in a bounded set. The GP is one of the Bayesian optimization algorithms. Bayesian optimization algorithms use a cheap probabilistic surrogate model to approximate the expensive error function. Consequently, we use the GP to optimize the hyperparameters of the CapsNet. Algorithm (1) shows the detailed steps of the COVID-19 CapsNet Detection Model.
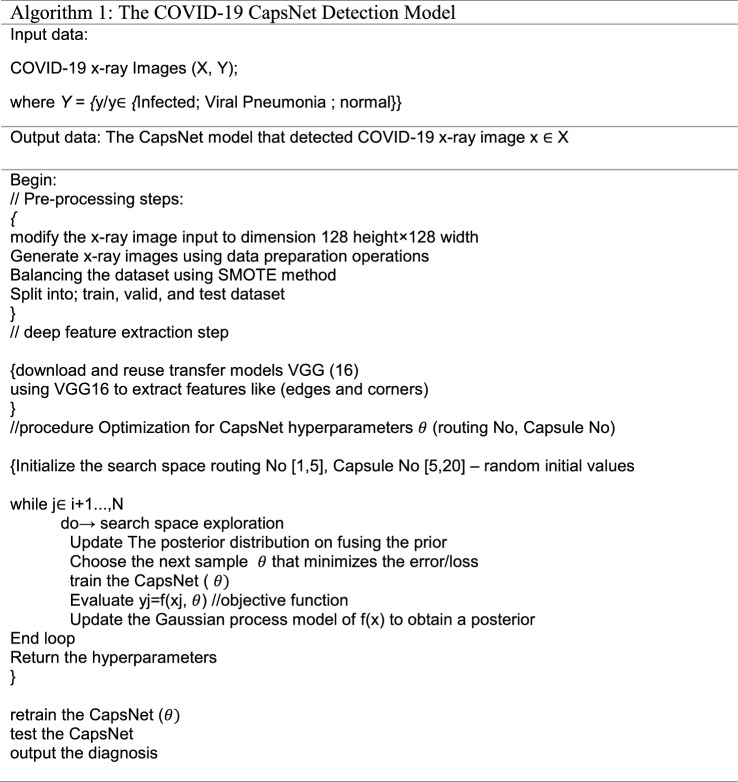


## Experimental results

The experiments were performed using tensor flow and Keras with a TPU google COLAB environment.

### Dataset

**Fig. 6:**
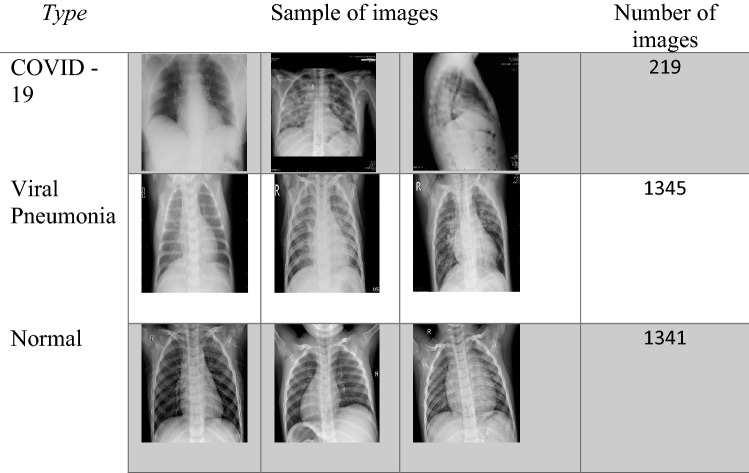
Sample images from the dataset

The COVID-19 Chest X-ray images used in this study are from the Italian Society of Medical and Interventional Radiology and are named the SIRM dataset. The dataset is hosted at Kaggle [37] and contains healthy instances, viral pneumonia, and COVID-19 patients. It contains 219 positive images for COVID-19, 1341 standard images, and 1345 images for viral pneumonia. The images are in portable network graphics file format with a 1024 × 1024 pixels resolution. The dataset is not balanced and may need some effort to balance before using the deep learning-based COVID-19 detection. Figure 6 shows samples of the training and testing images for the three classes. Panel Type (row-1) illustrates the COVID-19 infected, panel (row-2) shows viral pneumonia, and panel (row-3) illustrates healthy instances.

### Evaluation measures

The proposed model capacity is evaluated based on the accuracy (*Acc*), precision (*P*), recall (*R*), and *F1* score. Accuracy is the percentage of true predictions from all forecasts made, calculated by Eq. (11), precision assesses a model's ability to predict values for a specific category correctly, and it is calculated using Eq. (12), recall is calculated as the fraction of correctly classified positive patterns in Eq. (13). At the same time, F1 score is the weighted average of precision and recall calculated based on Eq. (14).

11 $${\text{Acc = (TP + TN)/(TP + FP + FN + TN)}}$$

12 $$P = {\text{TP}}/({\text{TP}} + {\text{FP}})$$

13 $$R = {\text{TP}}/({\text{TP}} + {\text{FN}})$$

14 $$FI = 2 \times (R \times P)/(R + P)$$

where *TP* = true positive samples, *TN* = true negative samples, *FP* = false positive samples, and *FN* = false negative samples [38].

### Experiments with different scenarios

Table 2 shows the initial setting parameters of the four CapsNets experiments.

**Table 2:** Parameter setting of all experiments

Experiments	Capsule dim	Routing#		Epochs#
CapsNet with the unbalanced data sets	10	5		10
CapsNet with balanced data sets based on class weight	10	5		20
CapsNet with balanced data sets based on SMOTE	10	5		20
CapsNet hyperparameters optimization with balanced data sets based on SMOTE	8	2		10

*Experiment Scenario (I)*: CapsNet-based pre-train VGG16 with the unbalanced data sets.

**Fig. 7:**
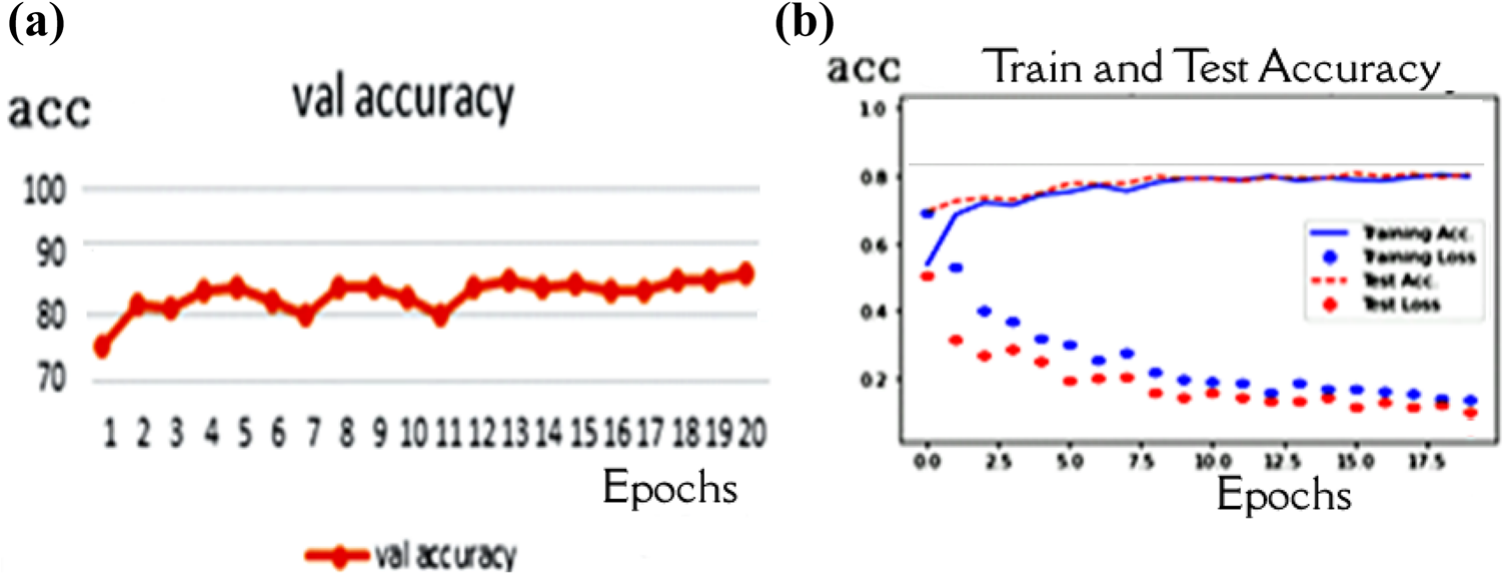
Performance of the model with the unbalanced data sets: **a** Validation accuracy **b** model performance during the training process

In the first experiment, the capsule network parameters were set as dim of capsule = 10 and routing = 5 after running the experiment with 20 epochs. The experiment achieved 89.93% accuracy, as shown in Fig. 7, which is unsatisfactory. This scenario did not consider the problem of unbalancing the data sets.

*Experiment Scenario (II)*: CapsNet-based pre train VGG16 with balanced data sets using class weight.

**Fig. 8:**
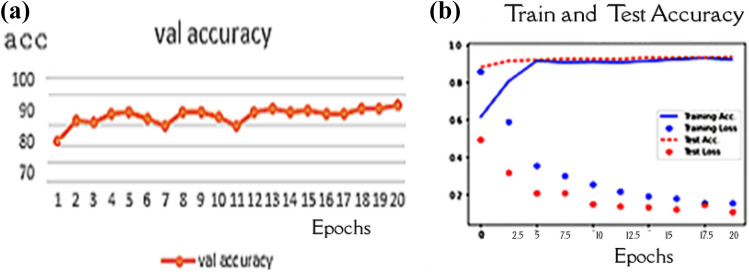
Performance of the model with balanced data sets based on class weight: **a** Validation accuracy **b** model performance during the training process

Figure 8 shows that this experiment is conducted with balanced data sets based on class weight strategy. The weight of the classes is 5.93089431, 0.67359187, and 0.74249364. The second experiment calculated the class weight for each class, and then executed the proposed model. Then, the Capsule network parameters were set as dim of capsules = 10 and the routing = 5. After running the experiment with 20 epochs, the accuracy is 94.46%.

*Experiment Scenario (III)*: Using SMOTE method, CapsNet-based pre-train VGG16 with balanced data sets.

**Fig. 9:**
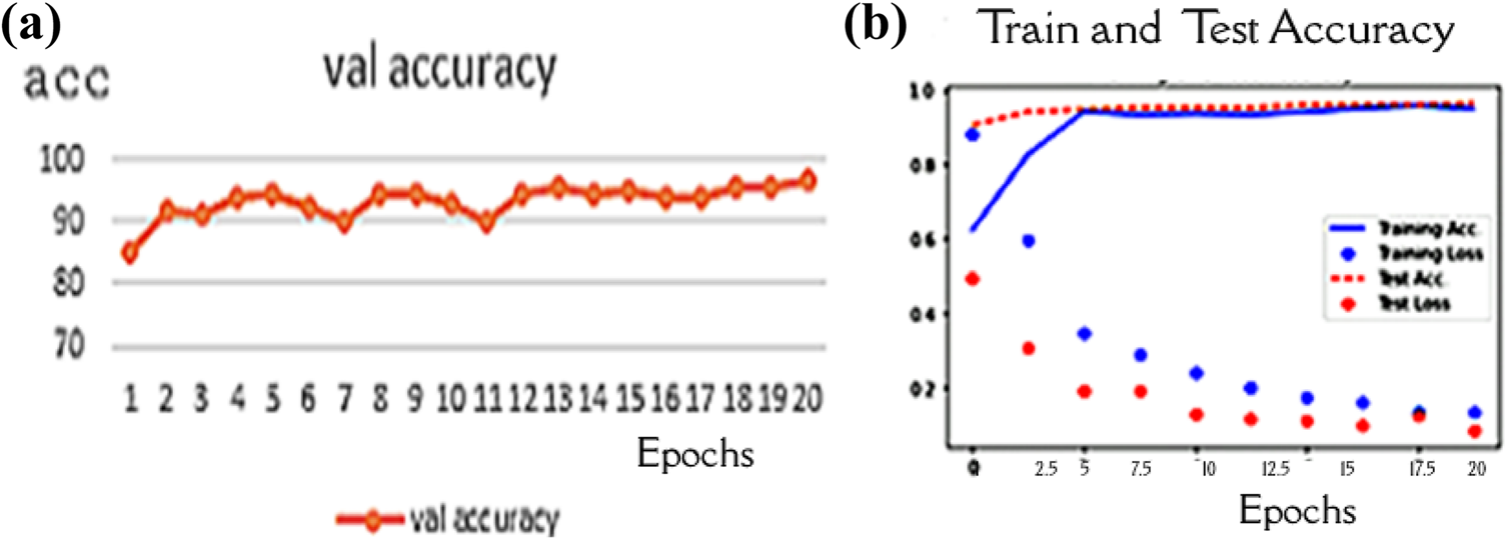
Performance of the model after using SMOTE: **a** Validation accuracy **b** model performance during the training process

The SMOTE method was used in this experiment to balance the dataset in the third experiment. This method regenerates the minority class to match the majority class, so the number of images in the dataset will be similar and equal to 1345. The over-sampling process took a long time, but it gave better performance. The Capsule network parameters were also set as dim capsules equal to 10 and the routing equal to 5 with 20 epochs. The overall test accuracy is 96.73% with the same hyperparameters of the Capsule, as shown in Fig. 9.

*Experiment Scenario (VI)*: CapsNet hyperparameters optimization with balanced data sets using SMOTE method.

CapsNet's hyperparameters were fine-tuned based on the Gaussian optimization algorithm. The fourth experiment ran an optimization process using the Gaussian method with 11 iterations, the minimum epochs for the GP in the Keras environment. In each iteration, the training epochs were only seven to save time. The best effect is achieved with two routings and eight dim for the capsules with only 10 epochs for each training cycle; refer to Table 3.

**Table 3:** Iteration based CapsNet optimzation results

iteration #	Routing	dim of capsules	Accuracy %
1	4	10	90.27
2	3	10	87.55
3	3	9	89.31
4	4	11	89.31
5	2	4	91.4
6	2	8	93.14
7	1	13	92.82
8	2	10	88.99
9	2	12	87.03
10	4	16	93
11	4	5	88.7

**Fig. 10:**
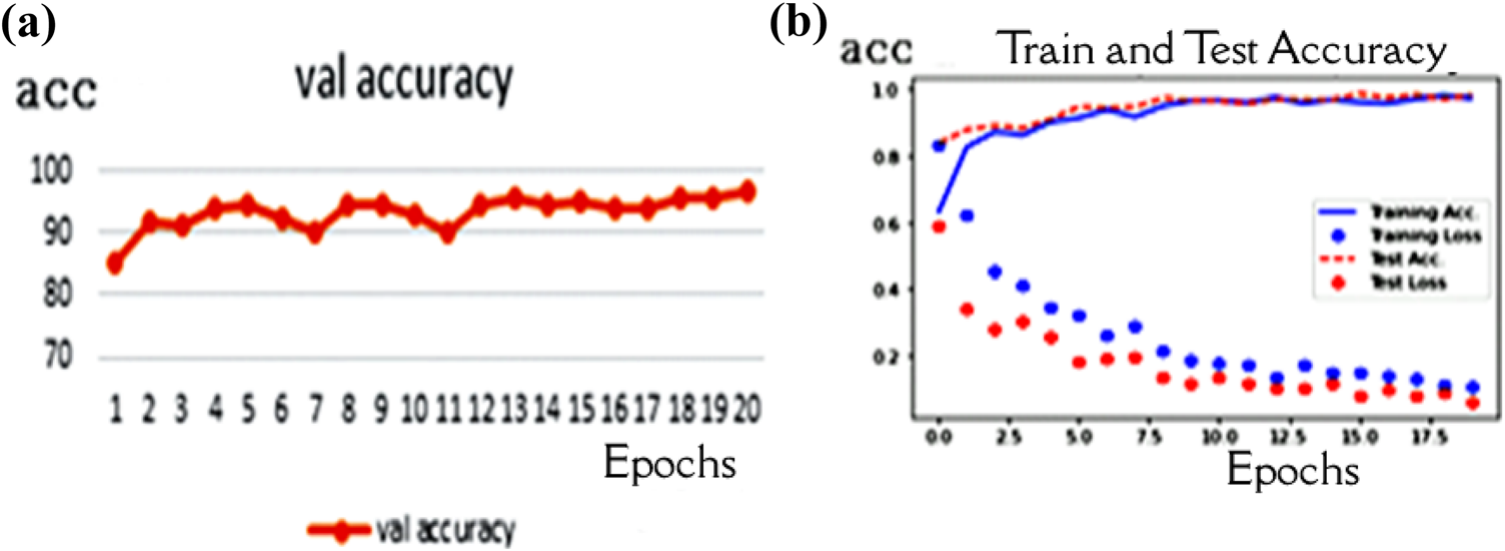
Performance of the model after capsule hyperparameters optimization: **a** Validation accuracy **b** model performance during the training process

After the optimization experiment, which has shown the best accuracy with routing = 2 and capsule dimension = 8, we executed the investigation with 20 epochs. The accuracy through the training is 96.58%, as shown in Fig. 10.

### Comparative analysis

The first comparative analysis was done among the four scenarios based on CapsNet. Table 4 describes each experiment's test accuracy, precision, recall, and F1 score. The Table shows that the proposed model has the highest F1 score. As we see from Table 4, the CapsNet hyperparameters optimization with balanced data sets based on class weight improves Recall and F1-score.

**Table 4:** Experimental scenarios comparative results

	Acc (%)	P	R	F1 score
CapsNet_VGG16 with imbalance data set	89.93	0.8379	0.9090	0.872
CapNet_VGG16 with balanced data by SMOTE	96.73	0.9718	0.9547	0.9631
CapsNet_VGG16 with balanced data by class weight (before optimization)	94.46	0.9525	0.9377	0.9400
CapsNet_VGG16 with balanced data by SMOTE (After optimization)	96.58	0.9652	0.9765	0.9708

The second comparative analysis is based on state of art [15–20, 22]. The proposed model's performance was compared with related work in terms of binary and multi classes problems. For the multiclass results obtained with different algorithms similar to the work we did in this paper, as shown in Table 5, we found that the proposed system has higher accuracy than all related work except [19]. In contrast, the best F1 score of the proposed system is better than all algorithms, including [19]. However, the proposed model has the best F1 score; it achieved 97.08%, while Nour et al. [19] have 96.7%, using the same dataset. Table 6 shows the training and testing running time within different epochs. At the same time, the complexity time is estimated as O(N^2 + Rou# + Caps#), where N is the number of Epochs, Rou# is the number of routing, and Caps# is the number of Capsule number.

**Table 5:** Comparison with related work

References	Dataset size	Class #	Performance	
			Accuracy %	F1-score
[15]	1144	Multi class	–	89
[20]	860	Binary class	89.3	90
[17]	196	Multi class	93.1	–
[16]	582	Binary class	98.061	98.551
		Multi class	91.329	91.73
[18]	50	Binary class	92.85	–
	455	Binary class	96.7	–
	603	Multiclass	93.07	–
[19]	2905	Multi class	98.97	96.7
[22]	Not mentioned	Binary class	98.08	96.51
		Multi class	87.02	87.02
The proposed model	2905	Multi class	96.58	97.08

**Table 6:** Running time on the training and testing phase

Training time (Epochs number)				Testing time
10 teration	15 iteration	10 iteration	5 iteration	5.61173 min
8.423 h	6.32222 h	4.22388 h	2.119166 h	

### Discussion and final notes

No single test can confirm or exclude a particular diagnosis in the medical field and diagnosis in general. All emerging diseases, especially developments in the behavior of Covid and changes that appear like the Micron strain, are rapidly evolving issues. Their treatment or diagnosis protocol will change over time and with the development of clinical expertise. Complete confirmation cannot be achieved in all cases unless a well-designed, multi-test diagnostic protocol is applied. We believe this study is a step toward helping clinicians make diagnoses with a high degree of accuracy and does not aim to rule out all other diagnostic tests for COVID-19 or replace them with X-rays. The current study aims to facilitate and expedite the analysis of chest X-rays taken during various diagnostic protocols for COVID-19. Especially now, after the systematic increase in the number of COVID-19 patients every day, it has become very difficult and universal for all medical personnel to perform the same high-quality analysis of chest X-rays 24/7. Therefore, automating certain steps of diagnostic protocols is necessary to maintain the integrity of diagnostic quality for medical field practitioners [23].

One of the advantages of using deep learning models is that feature selection is easy and does not require any requirements for selecting features manually. But in the case of medical applications, especially Covid-19, the selection of features by deep learning models is of great importance in evaluating and diagnosing diseases with high accuracy. However, doctors cannot explain them, so confidence in the results is uncertain. So, these features extracted from the Pre-trained CNN model (VGG16) need interpretations and explanations, so doctors and specialists can benefit from them. So, explanatory AI can assist clinical decision-makers in reacting to the challenges of a pandemic promptly.

## Conclusion and future work

A capsule consists of a collection of neurons, each reflecting an object's attribute. The direction of a capsule indicates an object's position, while the Capsule's length indicates the likelihood of the object's existence. To accomplish this, the capsule network uses routing algorithms to estimate the link strength between each Capsule. The connection linkages between capsules in successive levels describe part-whole relationships between the objects represented by the capsules.

This paper proposed a new COVID-19 detection model based on pre-trained CNN (VGG16) with CapsNet neural network. The model handles the imbalance and small data size based on SMOTE and the augmentation process. Four scenarios have been implemented, and the Gaussian optimization process to tune the hyperparameters of the Capsule neural networks has been used. The results show that the performance of the pre-trained with optimized CapsNet gets better results than the other scenarios implemented in this paper and gets better performance with the related work. The optimized CapsNet model achieved an accuracy rate of 96.58% and an F1- score of 97.08%, a competitive optimized model compared to other related models. Explain ability is receiving increasing attention in deep learning. Various ideas and tools are being developed to improve the annotation of deep learning models, an exciting direction toward eventual clinical acceptance of deep learning. Future work will use Explainable Artificial Intelligence (XAI), where doctors and people can understand the results of the solution. Also, since lung sounds represent a reliable marker of COVID-19 pneumonia, we will try to study both models (X-ray and lung sounds) in COVID-19.

## Data Availability

The data sets used in this paper are benchmark data and Kaggle [37].
